# The importance of the bacterial spectrum in the clinical diagnostics and management of patients with spontaneous pyogenic spondylodiscitis and isolated spinal epidural empyema: a 20-year cohort study at a single spine center

**DOI:** 10.1186/s12879-023-08946-x

**Published:** 2024-01-02

**Authors:** Mido Max Hijazi, Timo Siepmann, Ibrahim El-Battrawy, Percy Schröttner, Dino Podlesek, Gabriele Schackert, Tareq A Juratli, Ilker Y Eyüpoglu, Andreas Filis

**Affiliations:** 1 Department of Neurosurgery, Division of Spine Surgery, Technische Universität Dresden, Faculty of Medicine, and University Hospital Carl Gustav Carus, Fetscherstrasse 74, 01307 Dresden, Germany; 2 Department of Neurology, Technische Universität Dresden, Faculty of Medicine, and University Hospital Carl Gustav Carus, Fetscherstrasse 74, 01307 Dresden, Germany; 3 Department of Cardiology, Bergmannsheil University Hospital, Ruhr University Bochum, Bürkle de la Camp-Platz 1, 44789 Bochum, Germany; 4 Institute for Clinical Chemistry and Laboratory Medicine, Technische Universität Dresden, Faculty of Medicine, and University Hospital Carl Gustav Carus, Fetscherstrasse 74, 01307 Dresden, Germany; 5 Institute for Microbiology and Virology, Technische Universität Dresden, Faculty of Medicine, and University Hospital Carl Gustav Carus, Fetscherstrasse 74, 01307 Dresden, Germany

**Keywords:** Spondylodiscitis, Isolated spinal epidural empyema, Osteomyelitis, Bacterial spectrum, *Staphylococcus aureus*

## Abstract

**Background:**

Personalized clinical management of spondylodiscitis (SD) and isolated spinal epidural empyema (ISEE) is challenging due to limited evidence of microbiologic findings and their clinical impact during the clinical course of the disease. We aimed to characterize clinico-microbiological and imaging phenotypes of SD and ISEE to provide useful insights that could improve outcomes and potentially modify guidelines.

**Methods:**

We performed chart review and collected data on the following parameters: bacterial antibiogram-resistogram, type of primary spinal infection, location of spinal infection, source of infection, method of detection, clinical complications (sepsis, septic embolism, and endocarditis), length of hospital and intensive care unit (ICU) stay, relapse rate, and disease-related mortality in patients with proven pyogenic SD and ISEE treated surgically in a university hospital in Germany between 2002 and 2022.

**Results:**

We included data from 187 patients (125 SD, 66.8% and 62 ISEE, 33.2%). Gram-positive bacteria (GPB) were overall more frequently detected than gram-negative bacteria (GNB) (GPB: 162, 86.6% vs. GNB: 25, 13.4%, *p* < 0.001). Infective endocarditis was caused only by GPB (GPB: 23, 16.5% vs. GNB: 0, 0.0%, *p* = 0.046). Methicillin-susceptible *Staphylococcus aureus* was the most frequently isolated strain (MSSA: n = 100, 53.5%), occurred more frequently in the cervical spine compared to other bacteria (OB) (MSSA: 41, 41.0% vs. OB: 18, 20.7%, *p* = 0.004) and was most frequently detected in patients with skin infection as the primary source of infection (MSSA: 26, 40.6% vs. OB: 11, 16.7%, *p* = 0.002). *Streptococcus* spp. and *Enterococcus* spp. (SE: n = 31, 16.6%) were more often regarded as the cause of endocarditis (SE: 8, 27.6% vs. OB: 15, 11.4%, *p* = 0.037) and were less frequently detected in intraoperative specimens (SE: 19, 61.3% vs. OB: 138, 88.5%, *p* < 0.001). Enterobacterales (E: n = 20, 10.7%) were identified more frequently in urinary tract infections (E: 9, 50.0% vs. OB: 4, 3.6%, *p* < 0.001). Coagulase-negative *Staphylococci* (CoNS: n = 20, 10.7%) were characterized by a lower prevalence of sepsis (CoNS: 4, 20.0% vs. OB: 90, 53.9%, *p* = 0.004) and were more frequently detected in intraoperative specimens (CoNS: 20, 100. 0% vs. OB: 137, 82.0%, *p* = 0.048). Moreover, CoNS-associated cases showed a shorter length of ICU stay (CoNS: 2 [1–18] days vs. OB: 6 [1–53] days, median [interquartile range], *p* = 0.037), and occurred more frequently due to foreign body-associated infections (CoNS: 8, 61.5% vs. OB: 15, 12.8%, *p* = 0.008). The presence of methicillin-resistant *Staphylococcus aureus* (MRSA) prolonged hospital stay by 56 [24–58] days and ICU stay by 16 [1–44] days, whereas patients with *Pseudomonas aeruginosa* spent only 20 [18–29] days in the hospital and no day in the ICU 0 [0–5] days.

**Conclusions:**

Our retrospective cohort study identified distinct bacterial-specific manifestations in pyogenic SD and ISEE regarding clinical course, neuroanatomic targets, method of pathogen detection, and sources of infection. The clinico-microbiological patterns varied depending on the specific pathogens.

## Background

Primary spinal infections (PSI) account for 2–5% of all infectious bone diseases [1–3] and mainly include spondylodiscitis (SD) and isolated spinal epidural empyema (ISEE) [4–7]. The incidence of PSI was previously reported to be 0.2–3 cases per 100.000 persons per year [8–10], however current age-standardized incidence rate in Germany was estimated to be 30 per 250.000 persons per year based on data from the Federal Statistical Office (2015) [11].

Despite diagnostic and therapeutic improvements, PSI remains a challenging disease with a high mortality rate [12]. Microbiological analysis of the causative bacteria plays a key role in the diagnostic and treatment of PSI [13], thus effective antibiotic treatment depends on the correct identification of the underlying bacterial species and their antimicrobial susceptibility.

To verify an infection, at least two peripheral blood cultures and several biopsies should be submitted for microbiological diagnostics [14]. Bacterial detection rate in PSI is successful in 50–83% of cases [15, 16]. In previous studies, methicillin-susceptible *Staphylococcus aureus* (MSSA) was the most detected bacterium in PSI, followed by coagulase-negative *Staphylococci* (CoNS) [12, 17–21].

A better understanding of the clinico-microbiological differences between the various bacterial species would improve the treatment and outcome, thus affecting the complication rate and length of stay in the hospital and intensive care unit (ICU). We analyzed the bacterial spectrum in our cohort of 187 surgical patients and assessed its impact on clinical disease course to determine a reproducible phenotyping of a bacterial population in SD and ISEE.

## Methods

### Study design and patient data

**Fig. 1:**
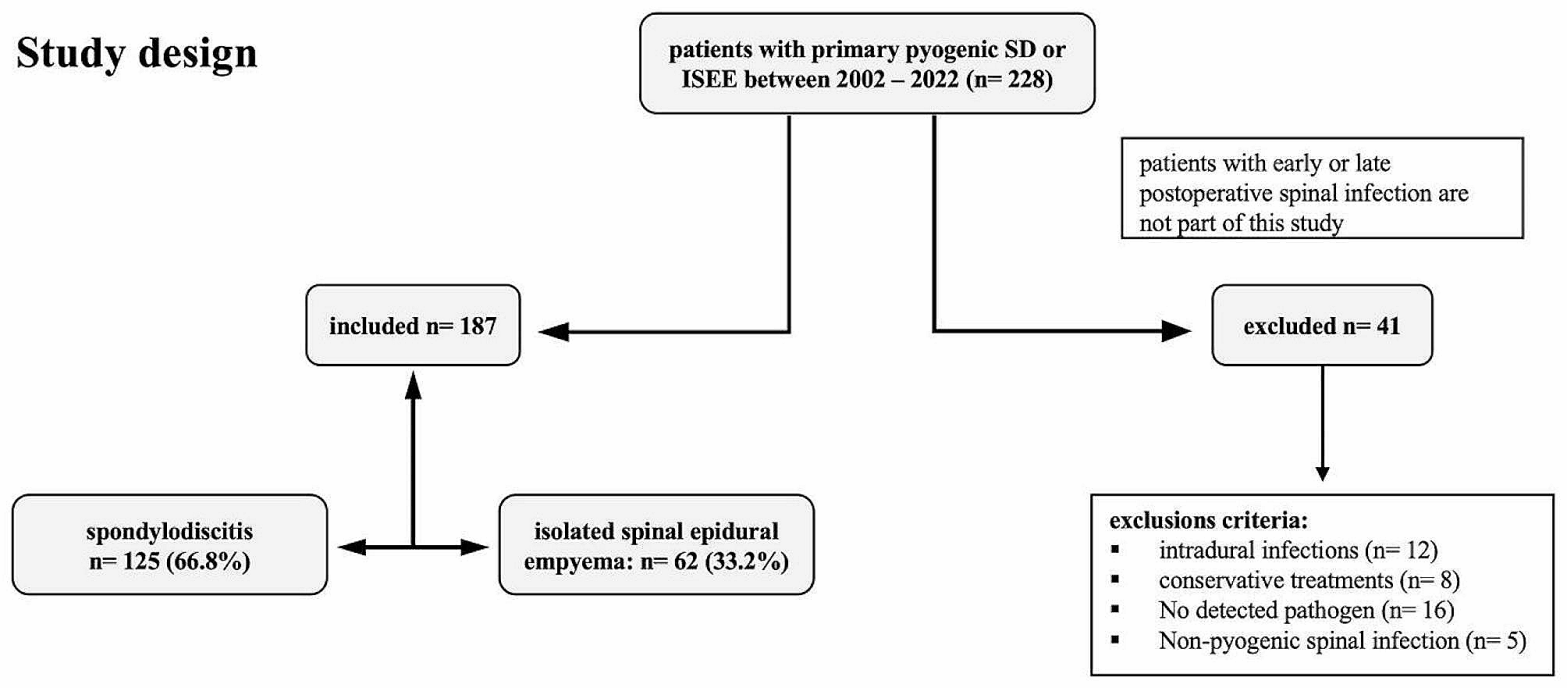
Study design: One hundred eighty-seven patients with proven pyogenic spondylodiscitis (SD: 125) and isolated spinal epidural empyema (ISEE: 62) were included in this study. Forty-one patients were excluded based on exclusion criteria

We performed a retrospective analysis of consecutive spontaneous PSI cases from 2002 to 2022 treated surgically at our Neurosurgery University Spine Center in Dresden, Germany. All patients with proven PSI who underwent surgical treatment were included. We excluded five cases with non-pyogenic pathogens, sixteen cases without any evidence of pathogens, eight patients with only conservative treatment, and twelve patients with intradural infection. A total of 187 patients were included in the analysis (Fig. 1).

The study was approved by the local ethics committee of the Carl Gustav Carus University Hospital in Dresden (Ref: BO-EK-17,012,022). Patients’ data were collected via the ORBIS system (ORBIS, Dedalus, Bonn, Germany) and neuroimaging studies through the IMPAX system (IMPAX, Impax Asset Management Group plc, London, UK).

Electronic medical records were first pseudonymized and then analyzed for various laboratory, microbiologic, histopathologic, clinical, and radiologic parameters. Relapse was defined as clinical, microbiological, and/or radiological progression during or after treatment up to the final follow-up of one year. We evaluated relapse rate, disease-related mortality, complication rate (sepsis [Soong, 2012 #49], septic embolism [Stawicki, 2013 #50], infective endocarditis [Barnett, 2016 #51]), and therapeutic modalities. Moreover, the detection rate of bacteria in blood culture, intraoperative specimen, and computed tomography (CT)-guided biopsy of psoas abscess was evaluated. To determine the source of infection in SD or ISEE, it is first necessary to understand the pathogenesis of spinal infections, which can begin in three different ways: direct contamination after surgery, adjacent spread from a neighboring soft tissue infection, or hematogenous spread from a distant source of infection such as pneumonia. If the spinal infection clearly emerged from one of these three routes and had the same pathogen, we called it the source of infection.

### Clinico-microbiological management

#### Diagnosis and treatment

SD and ISEE were diagnosed on the basis of clinical, laboratory, microbiological, and radiological findings (magnetic resonance imaging (MRI) and CT) according to the guidelines of the Infectious Diseases Society of America (IDSA) [14]. Clinical and laboratory parameters included back or neck pain, fever, leukocyte count, C-reactive protein (CrP), and Procalcitonin.

Depending on the clinical condition, at least two blood cultures were taken for microbiological diagnosis before antibiotic therapy was initiated, while some patients were initially treated with antibiotics in a peripheral hospital due to their severe clinical condition. In addition, tissue samples taken during open surgery and samples obtained by CT-guided biopsies were also used for microbiological analysis. We treated all patients surgically in different procedures depending on the location of the infection, clinical and laboratory findings, and severity of the bone defects.

Patients with hemodynamic instability, sepsis, septic shock, or progressive neurological symptoms underwent empiric antibiotic therapy (EAT) immediately after collection of blood cultures. On the other hand, patients with stable hemodynamic and neurological symptoms received targeted antibiotic therapy (TAT) after pathogen detection in the blood culture, image-guided biopsy, or open surgery. In the case of culture-negative spondylodiscitis (blood culture, image-guided biopsy, and surgery), we initiated an EAT. EAT was based on the suspected pathogen, suspected source of infection, clinical condition, epidemiologic risk, and local historical in vitro susceptibility data. In most cases, our therapy was a combination of vancomycin with ceftriaxone or vancomycin with piperacillin/tazobactam.

The first-line therapy in our clinic for MSSA was flucloxacillin (1. 5 − 2 g intravenously (IV) every (e) 4–6 h), β-haemolytic Streptococci or penicillin-susceptible Enterococci penicillin G (20–24 million units IV e 24 h), Enterobacteriaceae or Pseudomonas aeruginosa cefepime (2 g IV e 8–12 h), MRSA, CoNS, or penicillin-resistant Enterococci vancomycin (IV 15–20 mg/kg e 12 h with loading dose and monitoring of serum levels).

In patients with foreign body infections or osteosynthesis material (fixator as therapy), a combination such as flucloxacillin or vancomycin with rifampicin was used. Depending on the clinical condition, infection parameters and MRI findings, the IV-therapy was switched to an oral therapy for another 4 weeks.

#### Microbiological assessment

##### Blood culture

Both the aerobic and anaerobic blood culture flasks (BACTEC Plus Aerobic/F and Anerobic/F) were incubated for a maximum of 14 days in the BACTECTM FX blood culture machine (BD, Heidelberg, Germany). After cultural growth was indicated, a Gram staining was performed. Furthermore, an agar diffusion test was carried out using a Müller-Hinton agar (bioMérieux, Nürtingen, Germany) and test platelets (bestbion dx, Köln, Germany) for specific antibiotics to provide a provisional resistogram. Cultivation from aerobic culture was performed by plating sample on a Columbia blood agar (bioMérieux, Nürtingen, Germany), on chocolate agar (bioMérieux, Nürtingen, Germany), bile Chryosidine Glycerol Agar (Becton Dickinson, Heidelberg, Germany), Sabauroud glucose agar (Becton Dickinson, Heidelberg, Germany) and on Columbia CNA agar with 5% sheep blood (Becton Dickinson, Heidelberg, Germany). HCG agar (Sifin Diagnostics GmbH, Berlin, Germany) was used to detect anaerobic bacterial. It was incubated in a culture pot “AnaeroJar” (Becton Dickinson, Heidelberg, Germany) under absorption of oxygen using AnaeroGen (Oxoid, Wesel, Germany). The aerobic cultures were incubated at 37 degrees for 24 h. The HCG agar was incubated for 48 h. As soon as bacterial colonies were detectable, the species were identified using MALDI TOF MS (Bruker Daltonics GmbH, Bremen, Germany).

##### Tissue from CT-guided or intraoperative sampling

Tissue obtained intraoperatively or CT-guided was placed directly in the operating theatre in Schaedler boullions (bioMérieux, Nürtingen, Germany) and these were subsequently sent to the Institute of Medical Microbiology and Virology, Carl Gustav Carus University Hospital in Dresden for analysis. There, the boullions were first incubated at 37 degrees and examined for turbidity after 48 h. Once turbidity was detected, the culture suspension was plated on Columbia Blood Agar (bioMérieux, Nürtingen, Germany) and HCB Agar (bioMérieux, Nürtingen, Germany). Aerobic culture growth was checked for the first time after 24 h and anaerobic culture growth after another 48 h.

##### Antimicrobial susceptibility

The final antimicrobial susceptibility testing for aerobic bacterial species was performed using the VITEK 2 system (bioMérieux, Nürtingen, Germany) and the respective antimicrobial susceptibility test (AST) cards (bioMérieux, Nürtingen, Germany). The analysis was performed according to the guidelines of the manufacturer. Antimicrobial susceptibility profiles of anaerobic bacteria were generated using the ATB ANA System (bioMérieux, Nürtingen, Germany) or Gradient diffusion tests (bestbion, Cologne, Germany).

Bacterial species that are known to be of definite human pathogenic relevance have been included in the evaluation if they occur in already one sample. In contrast, low-pathogenic species (e.g., facultative pathogens) were only included in the evaluation if they were detected in at least two independent clinical samples. The results of the resistograms were then compared to assess whether two isolates were of the same origin or not.

#### Formation of groups

The detected bacteria were grouped in gram-positive bacteria (GPB) and gram-negative bacteria (GNB) and further subgrouped according to the taxonomic classification, specific resistance phenotypes or characteristic properties (MSSA, *Streptococcus* spp. and *Enterococcus* spp. (SE), CoNS, methicillin-resistant *Staphylococcus aureus* (MRSA), *bacillus* spp., *Pseudomonas aeruginosa*, Enterobacterales (E), and anaerobic bacteria). Furthermore, *Enterococcus* spp. and infections showing a polymicrobial spectrum were analyzed separately.

### Statistical analysis

Data were statistically analysed with the SPSS software package (SPSS Statistics 28, IBM, Armonk, New York, USA). Descriptive statistics were used, and categorical variables were adjusted by Fisher exact tests or chi-square tests. Numerical variables were analysed with Mann-Whitney U tests. All statistical tests were two-sided, and a p value p < 0.05 was considered statistically significant.

## Results

### Demographics and baseline characteristics

We included 187 patients (males: 120, 64.2% vs. females: 67, 35.8%, p < 0.001) aged 68 [23–90] y, median [interquartile range] with SD (125, 66.8%) and ISEE (62, 33.2%), of whom 162 (86.6%) had GPB and 25 (13.4%) had GNB (Table 1).

**Table 1:** Baseline characteristics

Baseline characteristics	N = 187	Percentage
Male	120	64.2%
Female	67	35.8%
Age	68 [23–90] y*	–
Spondylodiscitis	125	66.8%
Isolated spinal epidural empyema	62	33.2%
Gram-positive bacteria	162/187	86.6%
Gram-negative bacteria	25/187	13.4%
Surgery	187	100%
Blood cultures	187	100%
Psoas abscess	116	62.0%
CT-guided biopsy of Psoas	65/116	56.0%
Empiric antibiotic therapy (EAT)	122	65.2%
Targeted antibiotic therapy (TAT)	65	34.8%
Duration of intravenous antibiotics	4 [3–6] w*	–
Duration of total antibiotics	8 [6–12] w*	–
Sepsis	94/187	50.3%
Septic embolism	50/150	33.3%
Infective endocarditis with vegetation in TTE/TEE	23/161	14.3%
Relapse rate (clinical, microbiological, and/or radiological progression)	28/123	22.8%
Disease-related mortality	10/187	5.3%

CT: computer tomography, *median [interquartile range], w: week, y: years

Multiple blood cultures and intraoperative samples were taken from all patients, whereas 65 tappable patients (56.0%) out of 116 patients with psoas abscess (62%) underwent CT-guided biopsy. EAT was initially performed in 122 patients (65.2%) and later changed to TAT as soon as the underlying bacterial species was known, while 65 patients (34.8%) were treated directly with a TAT. Patients without antibiotic therapy or with an antibiotic-free period of more than 2 days formed the TAT group, whereas patients with ongoing antibiotic therapy or an antibiotic-free period of less than 2 days formed the EAT group. Patients received intravenous antibiotics for approximately 4 weeks (4 [3–6] w) and a total of 8 weeks antibiotics (8 [6–12] w). Sepsis was observed in 94 patients (50.3%), septic embolism in 50 patients (33.3%), and infective endocarditis with vegetation in echocardiography in 23 patients (14.3%). Twenty-eight patients (22.8%) relapsed, and ten patients (5.3%) died from the diseases and their complications (Table 1). To exclude septic embolism, 150 patients (80.2%) underwent radiological examination, 161 patients (86.1%) underwent TTE and TEE, and 123 patients (65.8%) underwent one-year clinical, laboratory, and radiological follow-up to exclude a relapse.

### Identified bacteria

**Fig. 2:**
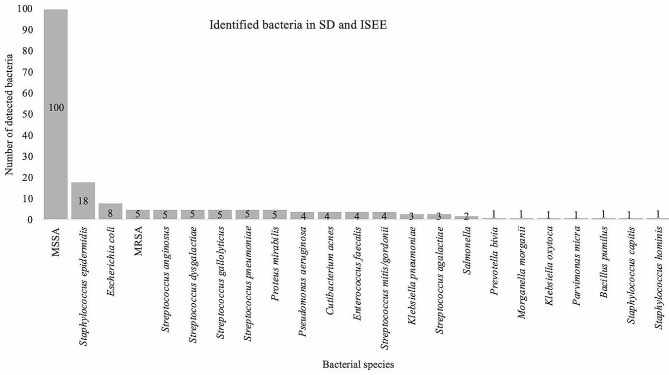
Identified bacteria in spondylodiscitis and isolated spinal epidural empyema: This figure shows the detected bacteria in patients with spondylodiscitis (SD) and isolated spinal epidural empyema (ISEE). MSSA: Methicillin-susceptible Staphylococcus aureus, MRSA: Methicillin-resistant Staphylococcus aureus

MSSA were the most commonly identified bacteria (100, 53.5%), followed by *S. epidermidis* (18, 9.6%) and *Escherichia coli* (8, 4.3%). MRSA, *Streptococcus anginosus*, *Streptococcus dysgalactiae*, *Streptococcus gallolyticus*, *Streptococcus pneumoniae*, and *Proteus mirabilis* were each identified in 5 cases (2.7%). *P. aeruginosa*, *Cutibacterium acnes*, *Enterococcus faecalis*, and *Streptococcus mitis/gordonii* were each observed in 4 cases (2.1%). *Klebsiella pneumoniae* and *Streptococcus agalactiae* were each isolated in 3 cases (1.6%). *Salmonella* spp. were found in 2 cases (1.1%). *Prevotella bivia*, *Morganella morganii*, *Klebsiella oxytoca*, *Parvimonas micra*, *Bacillus pumilus*, *Staphylococcus capitis*, and *Staphylococcus hominis* were each detected in 1 case (0.5%) (Fig. 2).

### Gram spectrum of bacteria

Bacterial spectrum was mainly dominated by GPB (GPB: 162, 86.6% vs. GNB: 25, 13.4%, p < 0.001). GPB were observed in 105 SD patients (64.8%) and in 57 ISEE patients (35.2%), while GNB were noted in 20 SD patients (80.0%) and 5 ISEE patients (20%) (p = 0.172). Endocarditis with vegetation in echocardiography occurred solely in GPB (GPB: 23, 16.5% vs. GNB: 0, 0.0%, p = 0.046). GNB showed a different distribution of primary infectious sources compared to GPB, especially more urinary tract infections (GPB: 4, 3.7% vs. GNB: 9, 40.9%, p < 0.001) (Table 2).

**Table 2:** Gram-positive and Gram-negative bacteria in spinal infections

Entities, complications, and primary source of infection (n = 187)	Gram-positive bacteria (n = 162, 86.6%)	Gram-negative bacteria (n = 25, 13.4%)	p-value*
SD	105/162 (64.8%)	20/25 (80.0%)	0.172
ISEE	57/162 (35.2%)	5/25 (20.0%)	
Sepsis	81/162 (50.0%)	13/25 (52.0%)	1.0
Septic embolism	46/128 (35.9%)	4/22 (18.2%)	0.142
Endocarditis	23/139 (16.5%)	0/22 (0.0%)	**0.046**
Relapse rate	22/103 (21.4%)	6/20 (30.0%)	0.394
Disease-related mortality	9 (5.6%)	1 (4.0%)	1.0
**Source of infection (129/187, 69.0%)**			
Hematogenous spread			**< 0.001** ^(1)^
Gastrointestinal tract infection	6/107 (5.6%)	3/22 (13.6)	
Respiratory tract infection (pneumonia)	15/107 (14.0%)	1/22 (4.5%)	
Urinary tract infection (urosepsis)	4/107 (3.7%)	9/22 (40.9%)	
Skin infection	34/107 (31.8%)	3/22 (13.6%)	
Foreign body-associated infection (joint replacements, venous access port, prosthetic heart valve, and screws elsewhere in spine)	20/108 (18.7%)	3/22 (13.6%)	
Odontogenic infection	5/107 (4.7%)	0/22 (0.0%)	
Retropharyngeal & prevertebral infection	4/107 (3.7)	2/22 (9.1%)	
Post-interventional			
Epidural administration such as spinal infiltration (facet joint, epidural, periradicular) or epidural catheter analgesia	19/107 (17.8%)	1/22 (4.5%)	

Bold values are significant results (p < 0.05) as indicated in the methods, SD: spondylodiscitis, ISEE: isolated spinal epidural empyema, *Fisher exact test

### Subgroups

Based on detailed review of the detected bacteria, we subgrouped the bacterial species according to the taxonomic classification, specific resistance phenotypes or characteristic properties. GPB excluding anaerobic bacteria (n = 157, 84.0%) were divided into MSSA (n = 100, 53.5%), SE (n = 31, 16.6%), CoNS (n = 20, 10.7%), MRSA (n = 5, 2.7%) and *Bacillus* spp. (n = 1, 0.5%), while GNB without anaerobic bacteria (n = 24, 12.8%) were grouped into E (n = 20, 10.7%) and *P. aeruginosa* (n = 4, 2.1%). Anaerobic bacteria (n = 6, 3.2%) included gram-positive *C. acnes* (n = 4, 2.1%) and *P. micra* (n = 1, 0.5%) and gram-negative *Prevotella bivia* (n = 1, 0.5%).

**Fig. 3:**
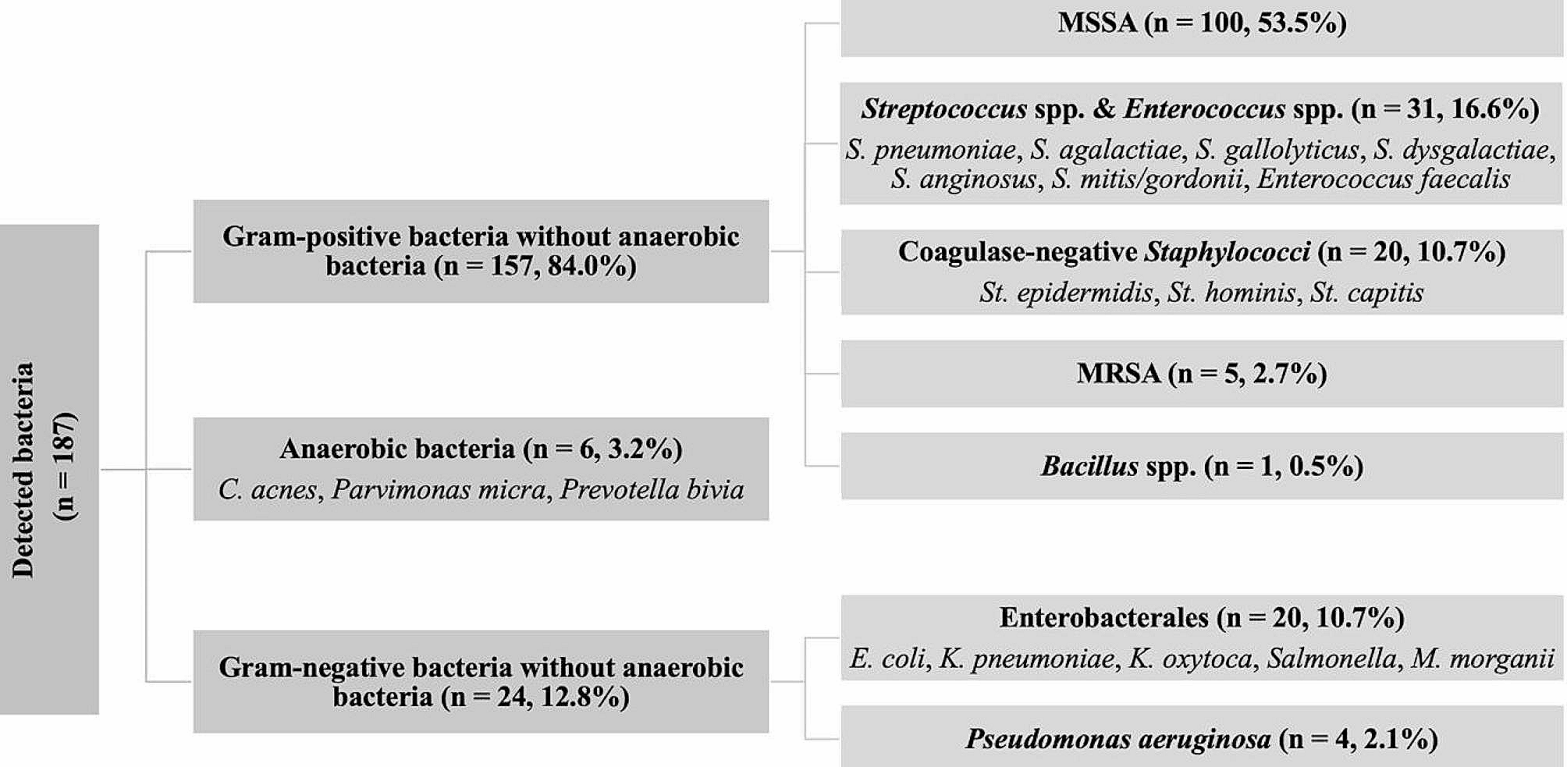
Bacterial subgroups in spondylodiscitis and isolated spinal epidural empyema: This figure demonstrates the subgroups of detected bacteria detected in our cohort. GPB: Gram-positive bacteria, GNB: Gram-negative bacteria. MSSA: Methicillin-susceptible *Staphylococcus aureus*, MRSA: Methicillin-resistant *Staphylococcus aureus*, St.: *Staphylococcus*, S.: *Streptococcus*, E.: *Escherichia*, K.: *Klebsiella*, M.: *Morganella*, C.: *Cutibacterium*

We analyzed and compared all subgroups with more than 20 cases, while small subgroups were evaluated separately (Fig. 3).

#### Methicillin-susceptible Staphylococcus aureus (MSSA)

MSSA were found less in SD and more in ISEE (SD: 57, 57.0% vs. ISEE: 43, 43.0%) compared to other bacteria (OB) (SD: 68, 78.2% vs. ISEE: 19, 21.8%, p = 0.003).

MSSA was found more in the cervical spine (CS) compared to other bacteria (OB) (MSSA: 41, 41.0% vs. OB:18, 20.7%, p = 0.004), but no differences were observed in samples from the thoracic spine (TS) (MSSA: 45, 45.0% vs. OB: 30, 34.5%, p = 0.178), and samples derived from the lumbar spine (LS) (MSSA: 64, 64.0% vs. OB: 59, 67.8%, p = 0.644).

The sources of infection in MSSA differed in distribution from OB (p < 0.001), particularly in relation to skin infections (MSSA: 26, 41.3% vs. OB: 11, 16.7%) and foreign body-associated infections (MSSA: 5, 7.9% vs. OB: 18, 27.3%) (Table 3).

**Table 3:** Clinical characteristics of methicillin-susceptible Staphylococcus aureus

Total n = 187	MSSA (100, 53.5%)	Other bacteria (87, 46.5%)	p-value
Sex	M: 63 (63.0%) F: 37 (37.0%)	M:57 (65.5%) F: 30 (34.5%)	0.761^(2)^
Age	67 [23–90] y	72 [47–89] y	0.052^(3)^
Spondylodiscitis	57 (57.0%)	68 (78.2%)	**0.003** ^(2)^
Isolated spinal epidural empyema	43 (43.0%)	19 (21.8%)	
Sepsis	56/100 (56.0%)	38/87 (43.7%)	0.108^(2)^
Septic embolism	29/79 (36.7%)	21/71 (29.6%)	0.389^(2)^
Endocarditis	13/86 (15.1%)	10/75 (13.3%)	0.824^(2)^
Relapse rate	10/64 (15.6%)	18/59 (30.5%)	0.056^(2)^
Disease-related mortality	6/100 (6.0%)	4/87 (4.6%)	0.753^(2)^
Length of hospital stay	31 [9-163] d	35 [10–165]	0.182^(3)^
Length of intensive care unit stay	8 [1–53] d	4 [1–46] d	0.093^(3)^
**Spinal localization**			
Cervical spine	41 (41.0%)	18 (20.7%)	**0.004** ^(2)^
Thoracic spine	45 (45.0%)	30 (34.5%)	0.178^(2)^
Lumbar spine	64 (64.0%)	59 (67.8%)	0.644^(2)^
**Bacterial detection via**			
Blood cultures	67 (67.0%)	51 (58.6%)	0.288^(2)^
Intraoperative specimens	87 (87.0%)	70 (80.5%)	0.238^(2)^
CT-guided biopsies (37/65)	23/41 (56.1%)	14/24 (58.3%)	1.0^(2)^
**Source of infection (129/187, 69.0%)**			
Hematogenous spread			**< 0.001** ^(1)^
Gastrointestinal tract infection	2/63 (3.2%)	7/66 (10.6%)	
Respiratory tract infection (pneumonia)	9/63 (14.3%)	7/66 (10.6%)	
Urinary tract infection (urosepsis)	3/63 (4.8%)	10/66 (15.2%)	
Skin infection	26/63 (41.3%)	11/66 (16.7%)	
Foreign body-associated infection (joint replacements, venous access port, prosthetic heart valve, and screws elsewhere in spine)	5/63 (7.9%)	18/66 (27.3%)	
Odontogenic infection	4/63 (6.3%)	1/66 (1.5%)	
Retropharyngeal & prevertebral infection	2/63 (3.2%)	4/66 (6.1%)	
Post-interventional			
Epidural administration such as spinal infiltration (facet joint, epidural, periradicular) or epidural catheter analgesia	12/63 (19.0%)	8/66 (12.1%)	

Bold values are significant results (p < 0.05) as indicated in the methods, MSSA: Methicillin-susceptible Staphylococcus aureus, CT: computed tomography, ^(1)^Chi-squared test, ^(2)^Fisher’s exact test, ^(3)^Mann–Whitney U test

#### *Streptococcus* spp. and *Enterococcus* spp. (SE)

*Streptococcus* spp. and *Enterococcus* spp. were found more often in SD but less frequently in ISEE (SD: 26, 83.9% vs. ISEE: 5, 6.1%) compared to OB (SD: 99, 63.5% vs. ISEE: 57, 36.5%, p = 0.036).

Endocarditis was found more frequently in the “SE subgroup” than in the “OB subgroup” (SE: 8, 27.6% vs. OB: 15, 11.4%, p = 0.037).

The diagnostic sensitivity for the “SE subgroup” was in intraoperative specimens lower compared to OB (SE: 19, 61.3% vs. OB: 138, 88.5%, p < 0.001), however no differences were observed in blood cultures (SE: 23, 74.2% vs. OB: 95, 60.9%, p = 0.221), and CT-guided biopsies (SE: 7/11, 63.6% vs. OB: 30/54, 55.6%, p = 0.745) (Table 4).

**Table 4:** Clinical characteristics of Streptococcus spp. und Enterococcus spp

Total n = 187	Streptococci & Enterococci (31, 16.6%)	Other bacteria (156, 83.4%)	p-value
Sex	M: 21 (67.7%) F: 10 (32.3%)	M: 99 (63.5%) F: 57 (36.5%)	0.688^(2)^
Age	72 [48–89] y	67 [23–90] y	0.190^(3)^
Spondylodiscitis	26 (83.9%)	99 (63.5%)	**0.036** ^(2)^
Isolated spinal epidural empyema	5 (16.1%)	57 (36.5%)	
Sepsis	16/31 (51.6%)	78/156 (50.0%)	1.0^(2)^
Septic embolism	12/27 (44.4%)	38/123 (30.9%)	0.184^(2)^
Endocarditis	8/29 (27.6%)	15/132 (11.4%)	**0.037** ^(2)^
Relapse rate	6/17 (35.3%)	22/106 (20.8%)	0.215^(2)^
Disease-related mortality	3/31 (9.7%)	7/156 (4.5%)	0.218^(2)^
Length of hospital stay	38 [10–165] d	32 [9-163] d	0.289^(3)^
Length of intensive care unit stay	7 [1–46] d	6 [1–53] d	0.944^(3)^
**Spinal localization**			
Cervical spine	23 (74.2%)	95 (60.9%)	0.221^(2)^
Thoracic spine	13 (42.0%)	62 (39.7%)	0.843^(2)^
Lumbar spine	25 (80.6%)	98 (62.8%)	0.064^(2)^
**Bacterial detection via**			
Blood cultures	23 (74.2%)	95 (60.9%)	0.221^(2)^
Intraoperative specimens	19 (61.3%)	138 (88.5%)	**< 0.001** ^(2)^
CT-guided biopsies (37/65)	7/11 (63.6%)	30/54 (55.6%)	0.745^(2)^
**Source of infection (129/187, 69.0%)**			
Hematogenous spread			0.344^(1)^
Gastrointestinal tract infection	3/24 (12.5%)	6/105 (5.7%)	
Respiratory tract infection (pneumonia)	4/24 (16.7%)	12/105 (11.4%)	
Urinary tract infection (urosepsis)	0/24 (0.0%)	13/105 (12.4%)	
Skin infection	5/24 (20.8%)	32/105 (30.5%)	
Foreign body-associated infection (joint replacements, venous access port, prosthetic heart valve, and screws elsewhere in spine)	6/24 (25.0%)	17/105 (16.2%)	
Odontogenic infection	0/24 (0.0%)	5/105 (4.8%)	
Retropharyngeal & prevertebral infection	1/24 (4.2%)	5/105 (4.8%)	
Post-interventional			
Epidural administration such as spinal infiltration (facet joint, epidural, periradicular) or epidural catheter analgesia	5/24 (20.8%)	15/105 (14.3%)	

Bold values are significant results (p < 0.05) as indicated in the methods, CT: computed tomography, ^(1)^: Chi-squared test, ^(2)^: Fisher’s exact test, ^(3)^: Mann–Whitney U test

#### Enterobacterales (E)

The sources of infection in Enterobacterales differed in distribution from OB (p < 0.001), particularly in relation to urinary tract infections (E: 9, 50.0% vs. OB: 4, 3.6%) (Table 5).

**Table 5:** Clinical characteristics of Enterobacterales

Total n = 187	Enterobacterales (20, 10.7%)	Other bacteria (167, 89.3%)	p-value
Sex	M: 12 (60.0%) F: 8 (40.0%)	M: 108 (64.7%) F: 59 (35.3%)	0.806^(2)^
Age	72 [54–83] y	68 [23–90] y	0.543^(3)^
Spondylodiscitis	16 (80.0%)	109 (65.3%)	0.218^(2)^
Isolated spinal epidural empyema	4 (20.0%)	58 (34.7%)	
Sepsis	10/20 (50.0%)	84/167 (50.3%)	1.0^(2)^
Septic embolism	3/19 (15.8%)	47/131 (35.9%)	0.117^(2)^
Endocarditis	0/18 (0.0%)	23/143 (16.1%)	0.078^(2)^
Relapse rate	6/17 (35.3%)	22/106 (20.8%)	0.215^(2)^
Disease-related mortality	1/20 (5.0%)	9/167 (5.4%)	1.0^(2)^
Length of hospital stay	33 [15–70] d	32 [9-165] d	0.629^(3)^
Length of intensive care unit stay	4 [1–17] d	7 [1–53] d	0.093^(3)^
**Spinal localization**			
Cervical spine	5 (25.0%)	54 (32.3%)	0.616^(2)^
Thoracic spine	5 (25.0%)	70 (41.9%)	0.227^(2)^
Lumbar spine	14 (70.0%)	109 (65.3%)	0.805^(2)^
**Bacterial detection via**			
Blood cultures	12 (60.0%)	106 (63.5%)	0.809^(2)^
Intraoperative specimens	16 (80.0%)	141 (84.4%)	0.535^(2)^
CT-guided biopsies (37/65)	1/4 (25.0%)	36/61 (59.0%)	0.307^(2)^
**Source of infection (129/187, 69.0%)**			
Hematogenous spread			**< 0.001** ^(1)^
Gastrointestinal tract infection	3/18 (16.7%)	6/111 (5.4%)	
Respiratory tract infection (pneumonia)	0/18 (0.0%)	16/111 (14.4%)	
Urinary tract infection (urosepsis)	9/18 (50.0%)	4/111 (3.6%)	
Skin infection	3/18 (16.7%)	34/111 (30.6%)	
Foreign body-associated infection (joint replacements, venous access port, prosthetic heart valve, and screws elsewhere in spine)	2/18 (11.1%)	21/111 (18.9%)	
Odontogenic infection	0/18 (0.0%)	5/111 (4.5%)	
Retropharyngeal & prevertebral infection	1/18 (5.6%)	5/111 (4.5%)	
Post-interventional			
Epidural administration such as spinal infiltration (facet joint, epidural, periradicular) or epidural catheter analgesia	0/18 (0.0%)	20/111 (18.0%)	

Bold values are significant results (p < 0.05) as indicated in the methods, CT: computed tomography, ^(1)^Chi-squared test, ^(2)^Fisher’s exact test, ^(3)^Mann–Whitney U test

#### Coagulase-negative Staphylococci (CoNS)

Sepsis (CoNS: 4, 20.0% vs. OB: 90, 53.9%, p = 0.004), and length of ICU stay (CoNS: 2 [1–18] d vs. OB: 6 [1–53] d, p = 0.037) were less frequent in CoNS than in OB. However, no differences were found in septic embolism (CoNS: 3, 20.0% vs. OB: 47, 34.8%, p = 0.387), endocarditis (CoNS: 2, 12.5% vs. OB: 21, 14.5%, p = 1.0), relapse rate (CoNS: 3, 18.8% vs. OB: 25, 23.4%, p = 1.0), disease-related mortality (CoNS: 0, 0.0% vs. OB: 10, 6.0%, p = 0.604), and length of hospital stay (CoNS: 34 [17–135] d vs. OB: 32 [9–165] d, p = 0.381).

The diagnostic sensitivity for detecting CoNS was in intraoperative specimens higher compared to OB (CoNS: 20, 100.0% vs. OB: 137, 82.0%, p = 0.048). However, no differences were observed in blood cultures (CoNS: 9, 45.0% vs. OB: 109, 65.3%, p = 0.089), and CT-guided biopsies (CoNS: 2/3, 66.7% vs. OB: 35/62, 56.5%, p = 1.0).

The sources of infection in CoNS differed in distribution from OB (p = 0.005), particularly in relation to skin infections (CoNS: 1, 7.7% vs. OB: 36, 31.0%) and foreign body-associated infections (CoNS: 8, 61.5% vs. OB: 15, 12.9%) (Table 6).

**Table 6:** Clinical characteristics of Coagulase-negative Staphylococci

Total n = 187	Coagulase-neg. Staphylococci (20, 10.7%)	Other bacteria (167, 89.3%)	p-value
Sex	M:14 (70.0%) F: 6 (30.0%)	M: 106 (63.5%) F: 61 (36.5%)	0.630^(2)^
Age	67 [47–82] y	69 [23–90] y	0.839^(3)^
Spondylodiscitis	15 (75.0%)	110 (65.9%)	0.464^(2)^
Isolated spinal epidural empyema	5 (25.0%)	57 (34.1%)	
Sepsis	4/20 (20.0%)	90/167 (53.9%)	**0.004** ^(2)^
Septic embolism	3/15 (20.0%)	47/135 (34.8%)	0.387^(2)^
Endocarditis	2/16 (12.5%)	21/145 (14.5%)	1.0^(2)^
Relapse rate	3/16 (18.8%)	25/107 (23.4%)	1.0^(2)^
Disease-related mortality	0/20 (0.0%)	10/167 (6.0%)	0.604^(2)^
Length of hospital stay	34 [17–135] d	32 [9-165] d	0.381^(3)^
Length of intensive care unit stay	2 [1–18] d	6 [1–53] d	**0.037** ^(3)^
**Spinal localization**			
Cervical spine	3 (15.0%)	56 (33.5%)	0.126^(2)^
Thoracic spine	7 (35.0%)	68 (40.7%)	0.810^(2)^
Lumbar spine	15 (75.0%)	108 (64.7%)	0.458^(2)^
**Bacterial detection via**			
Blood cultures	9 (45.0%)	109 (65.3%)	0.089^(2)^
Intraoperative specimens	20 (100.0%)	137 (82.0%)	**0.048** ^(2)^
CT-guided biopsies (37/65)	2/3 (66.7%)	35/62 (56.5%)	1.0^(2)^
**Source of infection (129/187, 69.0%)**			
Hematogenous spread			**0.005** ^(1)^
Gastrointestinal tract infection	0/13 (0.0%)	9/116 (7.8%)	
Respiratory tract infection (pneumonia)	1/13 (7.7%)	15/116 (12.9%)	
Urinary tract infection (urosepsis)	1/13 (7.7%)	12/116 (10.3%)	
Skin infection	1/13 (7.7%)	36/116 (31.0%)	
Foreign body-associated infection (joint replacements, venous access port, prosthetic heart valve, and screws elsewhere in spine)	8/13 (61.5%)	15/116 (12.9%)	
Odontogenic infection	0/13 (0.0%)	5/116 (4.3%)	
Retropharyngeal & prevertebral infection	0/13 (0.0%)	6/116 (5.2%)	
Post-interventional			
Epidural administration such as spinal infiltration (facet joint, epidural, periradicular) or epidural catheter analgesia	2/13 (15.4%)	18/116 (15.5%)	

Bold values are significant results (p < 0.05) as indicated in the methods, CT: computed tomography, ^(1)^Chi-squared test, ^(2)^Fisher’s exact test, ^(3)^Mann–Whitney U test

#### Anaerobic bacteria

Anaerobic bacteria were equally detected in SD and ISEE patients (SD: 3, ISEE: 3). Women were more frequently affected than men (women: 5, men: 1) and the median age was 80 [78–82] years. Two patients had septic embolism, and sepsis, but none had endocarditis or died, whereas one patient suffered a relapse. Patients spent 24.5 [13.5–34] days in the hospital and 1.5 [1–7.5] days on the ICU. All bacteria were identified only by intraoperative specimens. Three bacteria were found in the CS, one in the TS, and 2 in the LS. Retropharyngeal, and odontogenic infections were the primary of infections.

#### MRSA

The “MRSA subgroup” of 2 male and 3 female patients aged 81 [80–83] years was observed in 4 SD patients and one patient with ISEE. Sepsis was observed in 3 patients and endocarditis in 2 patients, but septic emboli did not occur. We found two relapses, but no in this subgroup. Prolonged hospital stay of 56 [24–58] days and ICU stay of 16 [1–44] days were recorded. In blood cultures and intraoperative specimens, four bacteria were detected, while two were detected in CT-guided biopsies. MRSA was diagnosed twice in the CS, once in the TS, and two times in the LS. Respiratory, gastrointestinal, and skin infections were the primary sources.

#### Pseudomonas aeruginosa

In three men and one woman aged 72 [71–75] years, *P. aeruginosa* caused three SD and one ISEE. Two patients had sepsis and one endocarditis, while no patient had a relapse or died. Patients spent 20 [18–29] days in the hospital and no day in the ICU 0 [0–5]. All bacteria were identified in intraoperative specimens and 2 of them in blood culture. Two patients underwent CT-guided biopsy of the psoas muscles, which was successful in one of them. Three bacteria were found in the TS and one in the CS, whereas LS was not affected. Respiratory and retropharyngeal infections were the source of the infections.

#### Polymicrobial Infections

In our cohort polymicrobial infections caused only spondylodiscitis and were documented in 8 patients. *Candida albicans* accounted for half of the polymicrobial infections and it was always associated with *S. aureus* (2 MSSA, 2 MRSA). *B. fragilis* was founded in infections with *P. aeruginosa*. *C. acnes* was present as a co-infection in MSSA infection. *S. epidermidis* was detected in a *S. anginosus* infection.

Therapy was adjusted according to the antibiogram. One patient died during treatment, and relapse was observed in 3 patients. The source of infection was pneumonia in two patients, gastrointestinal infection in 3 cases, per continuitatem spread of infection in two cases, and infection due to foreign body in one patient.

## Discussion

Our study provides insights into the bacterial spectrum and related clinical courses in SD and ISEE and identifies several bacterial group-related profiles. GPB were more frequently detected than GNB and exclusively caused infective endocarditis in our study. Previous studies showed that GPB was more common than GNB in ISEE and SD [8].

MSSA was the most common bacterium in SD and ISEE in our cohort, representing more than half of cases, which is consistent with the literature [12, 17–22]. In our study, MSSA occurred more frequently in ISEE than in SD, which was also observed by Stangenberg et al. [12]. MSSA occurred more frequently in CS than OB in our cohort, which previous studies have confirmed [12, 23–25]. Skin infections and epidural administrations such as infiltration therapies were the most source of infection in the “MSSA subgroup”. This is in line with studies showing that MSSA was common source of skin infections with spinal involvement [26–28].

The “SE subgroup” were the second most frequent bacterial subgroup in our study, causing 16.6% of infections, as reported in most studies [11, 29–31]. Nonetheless, some authors observed *S. epidermidis* being the second common causative bacteria [12, 22]. Endocarditis was more frequently found in the “SE subgroup”, in accordance with previous studies [11, 29]. *Streptococcus* spp. and *Enterococcus* spp. were detected more frequently in SD and less in ISEE, which has not been reported in literature yet. In accordance with the European diagnostic guidelines, we recommend first collecting blood cultures and then image-guided biopsy or open surgery, whereby the diagnostic sensitivity of intraoperative specimens in our study was lower in the “SE subgroup” (61.3%) and higher in the “CoNS subgroup” (100.0%) [Lazzeri, 2019 #48]. Other methods such as blood culture and CT-guided biopsy of psoas abscess showed no difference between the pathogen groups.

Infections with CoNS have increased recently due to the potential ability to biofilm formation and colonization in various surfaces, as well as the increasing use of medical implants [32]. One possible other cause would be the increasing sonication examination of foreign bodies. CoNS was associated with a shorter ICU stay and less sepsis and could always be isolated by intraoperative specimens, which was due to the detection of infected foreign bodies. In line with other studies, the source of infection in CoNS in our collective was frequently a foreign body-associated infection such as port infection [11, 33–35]. *S. epidermidis* is the second most common single bacteria after MSSA in our cohort (n = 18, 9.6%) and forms the fourth largest subgroup of SD and ISEE. Th previous studies reported similar results with a few exceptions [12, 22, 35, 36].

Enterobacterales were the main representatives of GNB and caused 10.7% of SD and ISEE in our collective. The literature data on the frequency of this subgroup in SD and ISEE varies widely [12, 22, 35]. Endocarditis was associated less frequently with Enterobacterales, in accordance with the literature data showing that endocarditis occurs in more than 80% of patients with GPB [11, 23]. Consistent with reported literature, the main source of infection in this subgroup was urinary tract infections [11, 35].

The “Anaerobic subgroup” could only be detected in intraoperative specimens, which showed the importance of this procedure. MRSA increased hospital length of stay to 56 days and ICU length of stay to 16 days, demonstrating the complexity of managing this infection, whereas patients with *P. aeruginosa* spent only 20 days in the hospital and no day in the ICU. Polymicrobial infections showed a complicated course and were half associated with *C. albicans*, which has not been previously investigated.

### Limitation and strengths of this study

Our study is limited by its retrospective design and a possible selection bias toward the more severe cases due to the high degree of specialization at our university center. Nevertheless, our data derived from detailed clinical, imaging, and microbiological state-of-the-art diagnostic assessment with high internal validity and a meaningful sample size Lastly, the generalizability of our observations is limited by the monocentric design of our study. The study spanned 20 years, and the number of patients per year is not large enough to make a conclusion about the distribution and transformation of the pathogens over a period of time.

## Conclusions

Our study shows that the bacterial groups in SD and ISEE have distinct bacterial-related patterns, which may help modify guidelines towards more tailored management.

GPB dominated the bacterial spectrum of SD and ISEE and were solely responsible for endocarditis. The most frequently isolated strain was MSSA, which was found more often in patients with skin infections and in the CS compared to other bacteria. Streptococci and enterococci were more frequently isolated in intraoperative specimens, were more frequently considered the cause of endocarditis, and were more often found in SD than in ISEE. CoNS resulted in less sepsis, shorter ICU stay, and was frequently associated with foreign body infection. Enterobacterales were the main representatives of GNB that did not cause endocarditis and were associated with urinary tract infections. Anaerobic bacteria could only be detected in intraoperative specimens in our study. In addition, MRSA increased hospital and ICU length of stay.

## Data Availability

The datasets supporting the conclusions of this article are included within the article.

## Abbreviations

PSI: Primary spinal infections
SD: Spondylodiscitis
ISEE: Isolated spinal epidural empyema
MSSA: Methicillin-Susceptible *Staphylococcus aureus*
CoNS: Coagulase-Negative *Staphylococci*
CT: Computed tomography
MRI: Magnetic resonance imaging
GPB: Gram-Positive bacteria
GNB: Gram-Negative bacteria
SE: *Streptococcus* spp. and *Enterococcus* spp
MRSA: Methicillin-Resistant *Staphylococcus aureus*
E: Enterobacterales
TTE: Transthoracic echocardiogram
TEE: Transesophageal echocardiogram
TAT: Empiric antibiotic therapy
EAT: Targeted antibiotic therapy
CS: Cervical spine
TS: Thoracic spine
LS: Lumbar spine
ICU: Intensive care unit
spp.: Species

## References

[1] Jevtic V (2004). Vertebral Infection. Eur Radiol.

[2] Khan IA, Vaccaro AR, Zlotolow DA (1999). Management of vertebral diskitis and osteomyelitis. Orthopedics.

[3] Tali ET (2004). Spinal Infections. Eur J Radiol.

[4] Alerhand S, Wood S, Long B, Koyfman A (2017). The time-sensitive challenge of diagnosing spinal epidural abscess in the emergency department. Intern Emerg Med.

[5] Babic M, Simpfendorfer CS (2017). Infections of the spine. Infect Dis Clin North Am.

[6] Davis DP, Wold RM, Patel RJ, Tran AJ, Tokhi RN, Chan TC, Vilke GM (2004). The clinical presentation and impact of diagnostic delays on emergency department patients with spinal epidural abscess. J Emerg Med.

[7] Shroyer SR, Davis WT, April MD, Long B, Boys G, Mehta SG, Mercaldo SF (2021). A clinical Prediction Tool for MRI in Emergency Department patients with spinal Infection. West J Emerg Med.

[8] Grammatico L, Baron S, Rusch E, Lepage B, Surer N, Desenclos JC, Besnier JM (2008). Epidemiology of vertebral osteomyelitis (VO) in France: analysis of hospital-discharge data 2002–2003. Epidemiol Infect.

[9] Krogsgaard MR, Wagn P, Bengtsson J (1998). Epidemiology of acute vertebral osteomyelitis in Denmark: 137 cases in Denmark 1978–1982, compared to cases reported to the National Patient Register 1991–1993. Acta Orthop Scand.

[10] Sapico FL, Montgomerie JZ (1979). Pyogenic vertebral osteomyelitis: report of nine cases and review of the literature. Rev Infect Dis.

[11] Herren C, Jung N, Pishnamaz M, Breuninger M, Siewe J, Sobottke R (2017). Spondylodiscitis: diagnosis and treatment options. Dtsch Arztebl Int.

[12] Stangenberg M, Mende KC, Mohme M, Kratzig T, Viezens L, Both A, Rohde H, Dreimann M (2021). Influence of microbiological diagnosis on the clinical course of spondylodiscitis. Infection.

[13] Gouliouris T, Aliyu SH, Brown NM (2010). Spondylodiscitis: update on diagnosis and management. J Antimicrob Chemother.

[14] Berbari EF, Kanj SS, Kowalski TJ, Darouiche RO, Widmer AF, Schmitt SK, Hendershot EF, Holtom PD, Huddleston PM, Petermann GW (2015). Executive Summary: 2015 Infectious Diseases Society of America (IDSA) Clinical Practice guidelines for the diagnosis and treatment of native vertebral osteomyelitis in adults. Clin Infect Dis.

[15] Pola E, Taccari F, Autore G, Giovannenze F, Pambianco V, Cauda R, Maccauro G, Fantoni M (2018). Multidisciplinary management of pyogenic spondylodiscitis: epidemiological and clinical features, prognostic factors and long-term outcomes in 207 patients. Eur Spine J.

[16] Sobottke R, Seifert H, Fatkenheuer G, Schmidt M, Gossmann A, Eysel P (2008). Current diagnosis and treatment of spondylodiscitis. Dtsch Arztebl Int.

[17] Appalanaidu N, Shafafy R, Gee C, Brogan K, Karmani S, Morassi G, Elsayed S (2019). Predicting the need for surgical intervention in patients with spondylodiscitis: the Brighton Spondylodiscitis score (BSDS). Eur Spine J.

[18] Gentile L, Benazzo F, De Rosa F, Boriani S, Dallagiacoma G, Franceschetti G, Gaeta M, Cuzzocrea F (2019). A systematic review: characteristics, Complications and treatment of spondylodiscitis. Eur Rev Med Pharmacol Sci.

[19] Loibl M, Stoyanov L, Doenitz C, Brawanski A, Wiggermann P, Krutsch W, Nerlich M, Oszwald M, Neumann C, Salzberger B (2014). Outcome-related co-factors in 105 cases of vertebral osteomyelitis in a tertiary care hospital. Infection.

[20] Pola E, Autore G, Formica VM, Pambianco V, Colangelo D, Cauda R, Fantoni M (2017). New classification for the treatment of pyogenic spondylodiscitis: validation study on a population of 250 patients with a follow-up of 2 years. Eur Spine J.

[21] Valancius K, Hansen ES, Hoy K, Helmig P, Niedermann B, Bunger C (2013). Failure modes in Conservative and surgical management of infectious spondylodiscitis. Eur Spine J.

[22] Adogwa O, Karikari IO, Carr KR, Krucoff M, Ajay D, Fatemi P, Perez EL, Cheng JS, Bagley CA, Isaacs RE (2014). Spontaneous spinal epidural abscess in patients 50 years of age and older: a 15-year institutional perspective and review of the literature: clinical article. J Neurosurg Spine.

[23] Donovan J, Skittrall JP, Moore T, Sargent C, Agranoff D, Llewelyn M (2016). An ageing population and changing UK bacteraemia profile may affect the characteristics and microbiology of infective spondylodiscitis. J Infect.

[24] Ghobrial GM, Franco D, Theofanis T, Margiotta PJ, Andrews E, Wilson JR, Harrop JS, Heller JE (2017). Cervical spondylodiscitis: presentation, timing, and Surgical Management in 59 patients. World Neurosurg.

[25] Shousha M, Heyde C, Boehm H (2015). Cervical spondylodiscitis: change in clinical picture and operative management during the last two decades. A series of 50 patients and review of literature. Eur Spine J.

[26] Cheung GYC, Bae JS, Otto M (2021). Pathogenicity and virulence of Staphylococcus aureus. Virulence.

[27] Klevens RM, Morrison MA, Nadle J, Petit S, Gershman K, Ray S, Harrison LH, Lynfield R, Dumyati G, Townes JM (2007). Invasive methicillin-resistant Staphylococcus aureus Infections in the United States. JAMA.

[28] Rasigade JP, Dumitrescu O, Lina G (2014). New epidemiology of Staphylococcus aureus Infections. Clin Microbiol Infect.

[29] Courjon J, Lemaignen A, Ghout I, Therby A, Belmatoug N, Dinh A, Gras G, Bernard L (2017). Group DTSs: pyogenic vertebral osteomyelitis of the elderly: characteristics and outcomes. PLoS ONE.

[30] Nolla JM, Ariza J, Gomez-Vaquero C, Fiter J, Bermejo J, Valverde J, Escofet DR, Gudiol F (2002). Spontaneous pyogenic vertebral osteomyelitis in nondrug users. Semin Arthritis Rheum.

[31] Pigrau C, Almirante B, Flores X, Falco V, Rodriguez D, Gasser I, Villanueva C, Pahissa A (2005). Spontaneous pyogenic vertebral osteomyelitis and endocarditis: incidence, risk factors, and outcome. Am J Med.

[32] Namvar AE, Bastarahang S, Abbasi N, Ghehi GS, Farhadbakhtiarian S, Arezi P, Hosseini M, Baravati SZ, Jokar Z, Chermahin SG (2014). Clinical characteristics of Staphylococcus epidermidis: a systematic review. GMS Hyg Infect Control.

[33] Jimenez-Mejias ME, de Dios Colmenero J, Sanchez-Lora FJ, Palomino-Nicas J, Reguera JM, de la Garcia J, Garcia-Ordonez MA, Pachon J (1999). Postoperative spondylodiskitis: etiology, clinical findings, prognosis, and comparison with nonoperative pyogenic spondylodiskitis. Clin Infect Dis.

[34] Legrand E, Flipo RM, Guggenbuhl P, Masson C, Maillefert JF, Soubrier M, Noel E, Saraux A, Di Fazano CS, Sibilia J (2001). Management of nontuberculous infectious discitis. Treatments used in 110 patients admitted to 12 teaching hospitals in France. Joint Bone Spine.

[35] Saeed K, Esposito S, Ascione T, Bassetti M, Bonnet E, Carnelutti A, Chan M, Lye DC, Cortes N, Dryden M (2019). Hot topics on vertebral osteomyelitis from the International Society of Antimicrobial Chemotherapy. Int J Antimicrob Agents.

[36] Bernard L, Dinh A, Ghout I, Simo D, Zeller V, Issartel B, Le Moing V, Belmatoug N, Lesprit P, Bru JP (2015). Antibiotic treatment for 6 weeks versus 12 weeks in patients with pyogenic vertebral osteomyelitis: an open-label, non-inferiority, randomised, controlled trial. Lancet.

